# The incidence of adverse events in an Italian acute care hospital: findings of a two-stage method in a retrospective cohort study

**DOI:** 10.1186/1472-6963-14-358

**Published:** 2014-08-27

**Authors:** Lorenzo Sommella, Chiara de Waure, Anna Maria Ferriero, Amalia Biasco, Maria Teresa Mainelli, Luigi Pinnarelli, Walter Ricciardi, Gianfranco Damiani

**Affiliations:** General Direction, San Filippo Neri Hospital Trust, Via G. Martinotti 20, Rome, 00135 Italy; Department of Public Health, Catholic University of Sacred Heart, L.go Francesco Vito 1, Rome, 00168 Italy; Medical Direction, San Filippo Neri Hospital Trust, Via G. Martinotti 20, Rome, 00135 Italy

**Keywords:** Clinical risk, Hospital, Ordinary admission, Charlson index, Comorbidity

## Abstract

**Background:**

The promotion of safer healthcare interventions in hospitals is a relevant public health topic. This study is aimed to investigate predictors of Adverse Events (AEs) taking into consideration the Charlson Index in order to control for confounding biases related to comorbidity.

**Methods:**

The study was a retrospective cohort study based on a two-stage assessment tool which was used to identify AEs. In stage 1, two physicians reviewed a random sample of patient records from 2008 discharges. In stage 2, reviewers independently assessed each screened record to confirm the presence of AEs. A univariable and multivariable analysis was conducted to identify prognostic factors of AEs; socio-demographic and some main organizational variables were taken into consideration. Charlson comorbidity Index was calculated using the algorithm developed by Quan et al.

**Results:**

A total of 1501 records were reviewed; mean patients age was 60 (SD: 19) and 1415 (94.3%) patients were Italian. Forty-six (3.3%) AEs were registered; they most took place in medical wards (33, 71.7%), followed by surgical ones (9, 19.6%) and intensive care unit (ICU) (4, 8.7%). According to the logistic regression model and controlling for Charlson Index, the following variables were associated to AEs: type of admission (emergency vs elective: OR 3.47, 95% CI: 1.60-7.53), discharge ward (surgical and ICU vs medical wards: OR 2.29, 95% CI: 1.00-5.21 and OR 4.80, 95% CI: 1.47-15.66 respectively) and length of stay (OR 1.03, 95% CI 1.01-1.04). Among patients experiencing AEs a higher frequency of elderly (≥65 years) was shown (58.7% vs 49.3% among patients without AEs) but this difference was not statistically significant. Interestingly, a higher percentage of patients admitted through emergency department was found among patients experiencing AEs (69.7% vs 55.1% among patients without AEs).

**Conclusions:**

The incidence of AEs was associated with length of stay, type of admission and unit of discharge, independently by comorbidity. On the basis of our results, it appears that organizational characteristics, taking into account the adjustment for comorbidity, are the main factors responsible for AEs while patient vulnerability played a minor role.

**Electronic supplementary material:**

The online version of this article (doi:10.1186/1472-6963-14-358) contains supplementary material, which is available to authorized users.

## Background

Adverse events (AEs), referred to as healthcare delivering, have been defined as injuries that are caused by medical management rather than the underlying disease [1] and which result in death, life threatening illness, disability at the time of discharge, admission to hospital, or prolongation of hospital stay [2–6]. Considering the complex nature of modern medical practice and the multitude of interventions delivered by now to each patient, a high rate of AEs might not be surprising, but if we only think of Hippocrates’ warning “Primum non nocere”, it is not acceptable at all. Moreover, the delivering of high standard quality of care would be a commitment for every healthcare organization because patient safety is a fundamental prerequisite to quality itself [7]. Nevertheless, AEs while receiving health care, even in technologically advanced hospital settings, are still so disturbingly common that, in 2009, the World Health Organization recognised patient safety as a serious public health issue affecting countries at all levels of development. Indeed, reducing the incidence of patient harm is a matter for everyone involved in health care [8, 9]. Since the 1970’s, epidemiological studies highlighted high rates of AEs experienced during hospital stay, ranging from 3.7% up to 36% [1, 10, 11]. Over the past 20 years, several studies, some of which nationwide, based on hospital records retrospective reviews, conducted in the USA [1, 5], Canada [12, 13], South America [14], Great Britain [6], Denmark [15], France [4], Germany [16, 17], Spain [18, 19], Sweden [20], Australia [21] and New Zealand [22, 23] have shown that the chance of a patient to experience an AE during hospitalization is still too high, ranging between 2.9% and 17%. Furthermore, it was noted that approximately half of the AEs were preventable [3, 16, 17]. Several studies have analyzed predictors of AEs [3, 4, 14, 17–19, 24, 25] such as emergency admission, surgical procedures, patient risk factors (age, gender, co morbidity etc.), length of stay, unit of discharge, organizational factors, human behaviors, and environmental causes. These predictors have been studied through different methods: cross sectional studies, prospective cohort, and retrospective cohort. Other studies [3, 14, 26] in order to evaluate how much co morbidity conditions could influence health outcomes, have used the Charlson Index.

This study is aimed at the following: 1) counting AEs occurring in an Italian acute care hospital by a 2-stage review of hospital charts; 2) assessing organizational predictors for AEs as well as individual risk factors; 3) taking into consideration the Charlson Index as a potential tool to control for confounding related to comorbidity.

## Methods

### Study design and setting

A retrospective cohort study was conducted to examine the incidence of AEs in an Italian acute care hospital. The clinical records included in the study were selected at random starting from the electronic archive of the hospital discharges. The sample included inpatients of all ages, if they stayed >24 h in the hospital and were discharged between the January 1, 2008 and December 31, 2008 (inclusive), day hospital discharges were not included. Inpatient care represented about 72% of the overall admissions in 2008. On the basis of these criteria, the sample comprised of 1,501 discharges, which represents the 7.3% of the overall inpatient admissions in 2008. All the hospital specialties were considered in the study in order to reflect the overall hospital practice. This study involved a two-stage sampling approach. Clinical records were analyzed with tools developed by Charles Vincent and colleagues [6]. The hospital clinical records were screened by two physicians (LP and AB) with experience in clinical risk management. The reviewers were trained by a theoretical-practical training course that lasted two and a half days. In the first stage of the review process (modular revision form 1 - RF1 -), the reviewers screened the records using 16 explicit screening criteria indicating potential AEs, adapted by those of Vincent et al. To test the validity of the process of screening, the two reviewers independently examined the first 10% of clinical records, and they compared assessments to see if they were in agreement with the identification of one or more criteria and the selection of potential AEs. The remaining 90% of clinical records was equally distributed by the two reviewers who reviewed them individually. If a record was screened as positive, the two physicians independently reviewed it. The uncertain cases were re-analyzed by reviewers, and if necessary, by a third reviewer (MTM). After screening, criteria-positive clinical records advanced toward the second-stage of the review and the modular revision form 2 (RF2) was completed. In this stage, each record that was positive for one or more criteria was reviewed independently by two physicians (LP and AB). After reviewing the clinical records, the two reviewers compared assessments to see if they agreed for the presence or absence of an AE. If the two reviewers did not agree, they discussed their differences, as in stage 1, and tried to reach an agreement. If the disagreement persisted after this comparison, the folder was submitted to the person responsible for the operational unit, and that person made the final decision. An additional file shows the Modular Revision Forms 1 and 2 in detail [see Additional file 1].

Regarding the association between incidence and organizational factors, four main indicators were taken into account: 1) length of stay, which was considered as an indicator of exposure; 2) type of admission which was an organizational factor linked to planning skills; 3) referral source which was an indicator of levels of coordination between the referral source of patient; 4) and unit of discharge, which was a structural indicator of the organizational context in which the AE occurred.)

No identifiable human data were used for this study. The data set used in the study is not openly available.

### Statistical analysis

Descriptive statistics were performed to describe a study of population characteristics. A univariable analysis was performed to assess the relationships between AEs and independent variables according to the following: age (<1 years; 1–15 years; 16–44 years; 45–64 years; ≥ 65 years), gender, residence (Lazio region or outside), nationality (Italian, foreign), marital status (single, married, separated, divorced, widowed), admission referral source (emergency department of the same hospital, other unit in the same hospital, different Hospital, specialist), admission type (elective, emergency or compulsory medical treatment), discharge unit (clinical ward, surgery ward, Intensive Care Unit – ICU), length of stay, and the Charlson Index. Chi square and Mann Whitney tests were used to perform the univariable analyses.

Variables whose p-value was less than 0.25 at the univariable analyses were entered a backward stepwise logistic regression model. An additive model was used to perform the analysis. Model goodness of fit was assessed through Hosmer and Lemeshow statistics. The results were shown in terms of Odds Ratio and 95% Confidence Intervals (95% CI).

In order to control for confounding related to comorbidity, the Charlson Index [27] was used. The Index was calculated according to the algorithms developed by Quan et al. [28], and by looking at Enhanced ICD-9-CM Coding in primary and secondary diagnoses. The Charlson Index was calculated by using STATA software version 9.0.

All of the other statistical analyses were conducted by using the statistical software SPSS version 12.0. Statistical significance was set at p = 0.05.

The paper follows the STROBE guidelines for reporting of observational studies [29]. An additional file shows the completed STROBE checklist [see Additional file 2].

### Ethics statement

Approval of the ethics committee was not required for the study because the Italian legislation (law 211/2003) attains to clinical research studies and does not provide statements on observational studies on routine collected, anonymous data. Data were extracted from routinely collected administrative databases and there was no need to obtain additional data from individual patients. The interventions under study were performed in ordinary or “natural” conditions, irrespective from the conduct of the present study. Because this was an observational retrospective study, patients had already been treated when the study protocol was written. Data linkage was performed by the team directly involved in patients’ care using numerical codes. For the present study, researchers had access only to an anonymous dataset, which ensured patients’ privacy. For these reasons, no personal informed consent to the present analysis was requested from study participants. The permission to medical records consultation was given by Medical Directions of hospitals involved in the study.

## Results

A total of 1,501 records were reviewed: 1,415 (94.3%) patients were Italian. The mean age of patients was 60 years (Standard Deviation: 19). One hundred and twenty-one records (8.1%) passed the first step of review process. Out of 121, 46 (3.3%) were judged to be AEs at the end of the second step review. Characteristics of all records are shown in Table 1.

**Table 1:** **Figure of selected inpatients ordinary admissions**

Variable	Absolute frequency	%
**Passage to RF2**		
*No*	1,380	91.9
*YES*	121	8.1
**Number of adverse events**	46	3.3
**Classes of age**		
*<1 year*	6	0.4
*1 - 15 years*	5	0.3
*16 - 44 years*	353	23.5
*45 - 64 years*	392	26.1
*≥ 65 years*	745	49.6
**Gender**		
*Male*	793	52.8
*Female*	708	47.2
**Lenght of stay for unit of discharge**		
**(median and interquartile range)**		
*Medical wards*	5 (7)	
*Surgical wards*	7 (10)	
*Intensive Care Unit*	18 (32)	
**Type of admission**		
*Elective*	641	42.8
*Mandatory compulsory obligatory*	9	0.6
*Emergency*	851	56.7
**Referral source**		
*Different mode from ordinary admission*	4	0.3
*Emergency Department of the same hospital*	832	55.4
*General Practicioner or Specialist*	636	42.4
*Transfered form other facility*	29	1.9
**Discharge unit**		
*Medical ward*	1,186	79.0
*Surgical ward*	292	19.5
*Intensive Care Unit*	23	1.5
**Residence**		
*Other Italian Region*	77	5.1
*Lazio Region*	1,424	94.9
**Citizenship**		
*Italian*	1,415	94.3
*Foreign*	86	5.7
**Marital status**		
*Not Married*	292	19.5
*Married*	939	62.6
*Separatoìed*	44	2.9
*Divorced*	38	2.5
*Widow*	188	12.5
**Age (mean and standard deviation)**	60 (19)	
**Charlson Index (median and interquartile range)**	0 (1)	

The univariable analysis showed that AEs were more common in patients with a longer length of stay, and in patients admitted to the hospital emergency. In fact, among all patients who experienced AEs, 36 (78.2%) were admitted to emergency, vs 10 (21.7%) admitted in election In patients coming from the emergency department of the same hospital as well as from other hospitals who experienced AEs, in fact among all patients experienced AEs, 32 (69.7%) were admitted from the Emergency Department, 10 (21.7%) from a specialists, and 4 (8.7%) came from other hospitals (Table 2).

**Table 2:** **Findings of univariable analysis**

Univariable analysis			
Variable	Adverse events no	Adverse events yes	p
**Age**			
<1 year	6 (0.4%)	0 (0%)	0.641
1 - 15 years	5 (0.3%)	0 (0%)	
16 - 44 years	346 (23.8%)	7 (15.2%)	
45 - 64 years	380 (26.1%)	12 (26.1%)	
≥ 65 years	718 (49.3%)	27 (58.7%)	
**Gender**			
Male	766 (52.6%)	27 (58.7%)	0.418
Female	689 (47.4%)	19 (41.3%)	
**Residence**			
Other Italian Region	77 (5.3%)	0 (0%)	0.109
Lazio	1378 (94.7%)	46 (100%)	
**Citizenship**			
Italian	1370 (94.2%)	45 (97.8%)	0.292
Foreign	85 (5.8%)	1 (2.2%)	
**Marital status**			
Not married	286 (19.7%)	6 (13.0%)	0.115
Married	914 (62,8%)	25 (54.3%)	
Separated	41 (2.8%	3 (6.5%)	
Divorced	36 (2.5%)	2 (4.3%)	
Widow	178 (12.2%)	10 (2.7%)	
**Lenght of stay**	6 (7)	15 (20)	<0.001
**(median and interquartile range)**			
**Type of admission (1499)**			
Elective	629 (43.3%)	10 (21.7%)	<0.001
Emergency	824 (56.7%)	36 (78.2%)	
**Referral source (1499)**			
*Different mode from ordinary admission*	4 (0.3%)	0 (0%)	<0.001
*Emergency Department of the same hospital*	800 (55.1%)	32 (69.7%)	
*General Practicioner or Specialist*	624 (42.3%)	10 (21.7%)	
*Transfered form other facility*	25 (1.7%)	4 (8.7%)	
**Discharge unit**			
Medical ward	1153 (79.2%)	33 (71.7%)	<0.001
Surgical ward	283 (19.5%)	9 (19.6%)	
Intensive Care Unit	19 (1.3%)	4 (8.7%)	
**Charlson Index**	0 (1)	0 (1)	0.99
**(median and interquartile range)**			

In regards to the unit of discharge, 4 (8.7%) patients, which experienced an AE, were discharged from ICU and 9 (19,6%) from surgical wards (Table 2). The length of stay was associated to AEs also (Table 2).

Patients aged at age 65 or older showed a higher frequency of AEs than those younger.

All these variables, together with age, gender, residence, marital status and the Charlson Index were entered in the multivariable regression model. The final model is shown in Table 3 and demonstrates that admission in emergency was associated to a higher risk for AE (OR 3.47, 95% CI 1.60-7.53) as well the length of stay (OR 1.03, 95% CI 1.01-1.04) and the discharge from surgery wards and ICU (OR 2.29, 95% CI 1.00-5.21 and 4.80, 95% CI 1.47-15.66 respectively).

**Table 3:** **Findings of multivariable analysis**

Variable	OR	CI (95%)
**Age**		
0 - 64 anni	1	
≥ 65 anni	1.40	0.75 – 2.61
**Gender**		
Male	1	
Female	0.77	0.41 -1.42
**Length of stay**	1.03	1.01 – 1.04
**Type of admission**		
Elective	1	
Emergency	3.47	1.60 – 7.53
**Discharge unit**		
Medical ward	1	
Surgical ward	2.29	1.00 – 5.21
ICU	4.80	1.47 – 15.66
**Charlson Index**	0.93	0.74 – 1.16

Hosmer and Lemeshow Test, Chi-square 5.368, p = 0.718.

## Discussion

This study showed that 3.3% of patients admitted to hospital experienced an AE. There is high variability of the phenomenon, proved by the higher values of prevalence showed in several studies, ranging from 3% to 17% in hospitalized patients [1, 2, 5, 6, 12, 15, 19–21, 23, 30, 31].

Our findings are in line with those reported in the Harvard Medical Practice Study [1] and in a recent work dealing with AEs in Dutch hospitals [17].

The incidence of AEs reported in this study was also lower than the overall 5.2% average which was yielded by a recent Italian multicenter study [32].

As far as risk factors for AEs, in our study, they were associated with length of stay, type of admission, referral source, and discharge unit.

With respect to length of stay, our study showed an association with AEs, in fact, for each incremental day of hospital stay, the related risk was increased by 3%. This result is in line with the international literature, and it might be explained by a prolonged exposure to risk factors [3, 12, 14, 18].

Regarding type of admission, among all patients who experienced AEs, 78.2% were admitted in emergency. This date, in contrast with the finding previously reported by Zegers et al. [3] could be due to the overcrowding of emergency patients. Indeed, as shown in the study of Ackroyd-Stolarz and colleagues, a prolonged stay in the emergency department is associated with an increased risk of any single AE (OR 1.03, 95% CI 1.004 to 1.05) [33]. Overcrowding in emergency departments is also a common phenomenon in Italian hospitals and could be mainly due to a shortage of available hospital beds, which results in prolonged emergency department stays for patients who need emergency admission [34].

Furthermore, Källberg et al. reported that the emergency department environment was described as complex, dynamic, and vulnerable to medical errors. In particular, the emergency department, in relation to communication, competence, triage, accessibility, and medication management, was identified as a possible risk area [35].

In regards to the referral source in the present work, there was no association with AEs, and that could be due to the adequate coordination mechanisms between the referral source of patients and the unit of discharge.

With respect to the discharge unit in our study, 71.7% of AEs were observed in medical wards even though a statistical significant association between the discharge unit and AE was shown only for surgical wards and ICU. As far as surgical wards this results are coherent with literature [12], and these could be mainly due to human factors [17]. As for ICU, several studies reported a high incidence of AEs ranging from 6.9 to 39.2% [36–44]. Moreover, the incidence of adverse events, according to Silberman et al., is proportional to the duration of ICU stay [45].

Relative to several intrinsic risk factors, the high frequency of AEs in patients admitted to ICU suggests that patient vulnerability could play a major role in generating AEs [3, 14, 18, 19, 30]. However, our study doesn’t show any association with comorbidity assessed by the Charlson Index.

The Charlson Index allowed an adjustment of the risk of AEs for comorbidity, and it relied on the evaluation of ICD-9 codes in primary and secondary diagnoses in the medical charts. In our study, we were able to conclude that, even if an underestimation of patient vulnerability was plausible, comorbidity did not have a great impact on the occurrence of AEs [14]. Notwinstanding further studies showed an association between the Charslon Index and AEs even if the methods for Charlson computation were different from ours [3]. Therefore severity of comorbidity could not be a main factor in developing AEs. On the other hand, organizational aspects could be responsible for the increased risk of AEs in particular wards. The study of Aranaz-Andrés reported a relation between the occurrence of AEs and the presence of extrinsic risk factors, such as urinary catheter, peripheral venous catheter, peripherally inserted central venous catheter, central venous catheter, parenteral nutrition, enteral nutrition, nasogastric tube, oesophagogastric percutaneous catheter, tracheostomy, mechanic ventilation or immunosuppression therapy [18]. Indeed, beyond patient vulnerability, the complexity of health-care services also appears to be associated with the development of AEs [18], and prevention also depends on the ability of the hospital environment to adapt to the variety of situations in which AEs occur [2]. Furthermore, a prolonged hospital stay, sure enough, could produce a higher exposure to organizational factors.

In view of what has been said, the use of a double-step tool for the detection of AEs, in association with a monitoring system of organizational factors adjusted for the Charlson index, could provide a useful contribution to clinical risk management.

Our study has some limitations. First of all, AEs were identified by means of a review process that relied on information enclosed in medical records, and this could produce a possible underestimation of AEs [18, 16]. The same reason could be responsible for a misclassification of the unit in which AEs occurred. In fact, our analysis accounted for the unit of discharge and the AEs that were attributed to it. Furthermore, it is probable that the AEs occurred in a different unit if the patient moved from one ward to another during the stay. Anyway it’s plausible that misclassification was not differential being only a dilution of risk possible.

Our study has several strengths. It was performed at the hospital level, which made it possible to have a thorough overview of AEs that occur in one year. Furthermore, the methodology used to identify AEs was based on a two-steps approach, which allowed strengthening the assessment. Two researchers were involved in the second step of the review process, which enabled the identification of AEs. The screening step was performed by two researchers on the first 10% of the clinical records with optimal agreement. Moreover, since the sampling was random, clinical records were not chosen with respect to their complexity. Another strength was related to the method used to account for comorbidity in the Charlson Index, which allowed us to tackle the problem of confounding that could interfere with the impact of organizational factors on AEs. Furthermore, Charlson index may be considered a good proxy of hospital case mix.

## Conclusions

In conclusion on the basis of our results, it appears that organizational characteristics, taking into account the adjustment for comorbidity, are the main factors responsible for AEs while patient vulnerability played a minor role.

Hospitals should implement risk management programs and address patients’ safety issues. From this viewpoint, it is fundamental to promote a continuous and timely evaluation of AEs with respect to their frequency, risk factors and costs.

## Electronic supplementary material

Additional file 1: **Modular Revision Forms 1 and 2.** The Modular Revision Forms are tools, adapted by those of Vincent et al. [6], used by reviewers to screen clinical records to detect AEs. (PDF 81 KB)

Additional file 2: **STROBE checklist.** STROBE checklist for reporting observational research, filled in relation to the present study. (PDF 150 KB)
