# Supplementary material for: The incidence of adverse events in an Italian acute care hospital: findings of a two-stage method in a retrospective cohort study
Source: BMC Health Serv Res. 2014 Aug 27;14:358. doi: 10.1186/1472-6963-14-358 (PMC4155122; doi:10.1186/1472-6963-14-358)
Supplement: Supplementary file 1 — Additional file 1: Modular Revision Forms 1 and 2. The Modular Revision Forms are tools, adapted by those of Vincent et al. [6], used by reviewers to screen clinical records to detect AEs. (PDF 81 KB) [file 12913_2014_3457_MOESM1_ESM.pdf]

REVIEW FORM 1 (RF1)

1. Was the patient taking any drug?
2. Does the patient have any comorbidity?
3. New unplanned re-admission within 21 days of discharge
4. Injury/fall during the hospital stay
5. Allergic reaction
6. Unplanned transfer from general care to intensive care or higher dependency
7. Unplanned transfer to another acute care hospital
8. Unplanned return to the operating room during the hospital stay
9. Complications during surgery, invasive procedures or delivery
10. Other complications including myocardial infarction, stroke, pulmonary embolism
11. Onset of new neurological deficit
12. Unexpected death
13. Cardio-respiratory arrest
14. Injury or complications related to abortion, labour and delivery including neonatal complications and low Apgar score
15. Hospital acquired infection or sepsis
16. Any other undesirable outcomes (not covered by any of the other criteria)

REVIEW FORM 2 (RF2)

An adverse event has to fulfil all three criteria:
1. an unintended injury or complication
2. temporary or permanent disability and/or increased length of stay or death
3. caused by health care management
